# Examining subjective well-being during pregnancy and its association with pregnancy intendedness among women in Nigeria: A population-based cross-sectional multilevel study

**DOI:** 10.1017/gmh.2024.99

**Published:** 2024-10-21

**Authors:** Otobo I. Ujah, Biodun N. Olagbuji, Russell S. Kirby

**Affiliations:** 1 College of Public Health, University of South Florida, Tampa, FL, USA; 2 Department of Obstetrics and Gynecology, Federal University of Health Sciences, Otukpo, Nigeria; 3 Department of Obstetrics and Gynecology, Ekiti State University, Ado-Ekiti, Nigeria

**Keywords:** pregnancy intention, life satisfaction, happiness, optimism, subjective well-being

## Abstract

In this study, we examined the patterns of subjective well-being (SWB) measures among pregnant women and quantified the extent to which pregnancy intendedness is associated with low SWB measures during pregnancy. We analyzed data from the 2021 Nigeria Multiple Indicator Cluster Survey comprising 3,491 pregnant women. The associations between pregnancy intention and low SWB measures (unhappiness, low life satisfaction [LS] and diminished optimism) were determined by fitting series of multilevel logistic regression models with random intercepts. Among pregnant women in our sample 20%, 37.5% and 9.6%, reported being unhappy, experiencing low LS and having diminished optimism, respectively. However, we found no significant association between pregnancy intention and being unhappy (mistimed: adjusted odds ratio [aOR] = 1.19, 95% CI = 0.88–1.60; unwanted: aOR = 1.16, 95% CI = 0.71–1.91), experiencing low LS (mistimed: aOR = 1.07, 95% CI = 0.83–1.37; unwanted: aOR = 1.06, 95% CI = 0.69–1.65) and having diminished optimism (mistimed: aOR = 1.22, 95% CI = 0.82–1.82; unwanted: aOR = 1.07, 95% CI = 0.56–2.04). Findings from the study suggest that pregnant women in Nigeria who reported having either a mistimed or unwanted pregnancy were just as likely to report being unhappy, experience low LS and have diminished optimism as women whose pregnancy was intended.

## Impact statement

Empirical studies investigating the effect of unintended pregnancy on women’s subjective well-being (SWB) are lacking. This study examining the association between maternal pregnancy intention and reports of low life satisfaction, unhappiness and diminished optimism during pregnancy aims to extend current literature on critical factors influencing maternal quality of life during pregnancy. By conducting this population-based multilevel cross-sectional analysis, we seek to provide valuable insights into the relationship between pregnancy intention and low SWB during pregnancy, thereby contributing to the development of targeted interventions and support systems to enhance the psychological health of expectant mothers. The findings from this study have the potential to inform healthcare policies and practices aimed at promoting positive prenatal experiences and improving maternal and child health outcomes. Furthermore, this research makes the case for longitudinal studies and research employing qualitative research methods to explore the relevant and potential pathways linking women’s pregnancy intentions and their psychological well-being in the perinatal period.

## Introduction

Reducing the burden of unintended pregnancy is an important public health priority. However, despite reported declines in the burden of unintended pregnancy globally, recent estimates suggest that between 2015 and 2019, nearly one in two pregnancies worldwide were unintended, being either mistimed or unwanted (Bearak et al., 2020). During this period, approximately 42% of pregnancies in sub-Saharan Africa were unintended, corresponding to a rate of 91 per 1,000 women (Bearak et al., 2020).

Unintended pregnancy represents a major reproductive health concern in Nigeria, as in many sub-Saharan African countries, considering that most cases of unintended pregnancies are resolved by having abortions, albeit unsafe (Sedgh et al., 2007; Bearak et al., 2022). This is largely due to the restricted access to safe and legal abortion services in these contexts (Bearak et al., 2020). For example, research conducted in Nigeria in 2012 revealed that approximately 56% of unintended pregnancies among women aged 15–49 were resolved through abortion (Bankole et al., 2015). More recent estimates from a study that used a social network-based measurement approach revealed an adjusted abortion incidence rate for the first confidants of 35.1 per 1,000 women of reproductive age in Nigeria over a 1-year period (Bell et al., 2020). Unintended pregnancies have been associated with poor physical and mental health outcomes for women, including reduced quality of life (Yazdkhasti et al., 2015; Yeatman and Smith-Greenaway, 2018; Bearak et al., 2020; Jang et al., 2021; Khan et al., 2022; Hobby et al., 2023). Evidence from observational studies have shown that unintended pregnancy, controlling for other factors, may independently increase the risk of maternal prenatal and postpartum depression (Faisal-Cury et al., 2017; Tasnim et al., 2021; Muskens et al., 2022; Blondel et al., 2023). This, in turn, may have negative implications for the well-being of the fetus, neonate and infant. Therefore, reproductive and childbearing decisions, together with its timing, are likely to not only impact the health and well-being of women but also that of their offsprings (Estinfort et al., 2022; Blondel et al., 2023), thereby underscoring the need for interventions to mitigate unintended pregnancy.

At the core of the 2030 sustainable development agenda is the goal of ensuring healthy lives and promoting individual well-being. An important aspect of well-being is self-perceived or subjective well-being (SWB), a cognitive process that reflects the appraisal of an individual’s quality of life (Helliwell and Barrington-Leigh, 2010; McDowell, 2010; Steptoe et al., 2015; Diener et al., 2017, 2018). This multidimensional construct, broadly categorized as evaluative (satisfaction with life), hedonic (happiness, sadness and anger) or eudaimonic (meaning and purpose of life) (Steptoe et al., 2015; Diener et al., 2017, 2018), well-being may play an important role in improving our understanding of how individuals and society function as a whole.

While health and well-being are theoretically distinct concepts, several studies have demonstrated a bidirectional relationship between the two constructs (Miret et al., 2014; Steptoe et al., 2015; Tran et al., 2017). Specifically, improved health has been shown to be associated with greater SWB (Ngamaba et al., 2017). On the other hand, Kushlev et al. (2020) also demonstrated that life satisfaction (LS) and positive affect are independently associated with health behavior. An understanding of this relationship is important as pregnancy intention impacts both maternal physical and mental health, which, in turn, influences and can also be influenced by the woman’s overall well-being (Diener et al., 2018; Hill et al., 2019).

Despite a growing interest among scholars in understanding the determinants and consequences of SWB, there remains a paucity of research focused on understanding the relationships between women’s pregnancy intention and subjectively oriented well-being measures especially in the peripartum period (Hardee et al., 2004; Herd et al., 2016). A recent systematic mapping review revealed that only eight studies to date have reported estimates of the association between pregnancy intention and SWB (Hill et al., 2019). Furthermore, the few existing studies examining the link between pregnancy intention and well-being have primarily operationalized pregnancy intention as either intended or unintended (Blake et al., 2007; Pishgar et al., 2016; Ali et al., 2023). However, this approach potentially obscures the differential effects of mistimed and unwanted pregnancies on SWB (Herd et al., 2016; Zimmerman et al., 2023), potentially leaving gaps in our underestimating of the complex relationships between pregnancy intention and SWB.

In this study, we examine whether measures of SWB among women reporting their pregnancy as unintended (i.e., mistimed or unwanted) differ from those reporting their pregnancy as intended, after controlling for individual-/household- and contextual-level characteristics. The purpose is to understand and quantify the extent to which unintended pregnancy is linked with happiness, LS and optimism during pregnancy. We hypothesize that women who reported their current pregnancy as either mistimed or unwanted would have a higher odds of reporting being unhappy, experiencing low LS and having diminished optimism compared to women reporting their pregnancy as intended.

## Methods

This cross-sectional analysis used population-level data from the sixth round of the Multiple Indicator Cluster Surveys (MICS6) conducted in Nigeria in 2021. The MICS is a nationally representative survey that uses a multistage stratified cluster sampling to collect data on sociodemographic and health indicators across various population groups, including households, children (aged 0–5 years), women aged 15–49 years and men aged 15–49 years. The survey was carried out by the National Bureau of Statistics (NBS) in Nigeria with support from UNICEF. The primary sampling strata consisted of states, and household sampling proceeded in two stages. Within each stratum, a predetermined number of census enumeration areas (EAs) were systematically selected based on probability proportional to their size. Thereafter, household listings were conducted within each selected EA, from which a systematic sample of 20 households was drawn per EA. Detailed information on the survey’s sampling design and data collection methods are documented elsewhere (NBS and UNICEF, 2022).

We limited our analysis to a sample of 3,565 women aged 15–49 years who reported being pregnant at the time of the survey. Observations missing data on pregnancy intention (*n* = 27), measures of LS (*n* = 1), happiness (*n* = 5), optimism (*n* = 18) and on confounding variables (marital status, *n* = 1; insurance, *n* = 17) were excluded. The final sample included 3,491 pregnant women within 1,316 clusters across the 37 strata. However, as the sample in the Nigeria MICS6 is not self-weighting (NBS and UNICEF, 2022), we applied the women’s sample weights included in the women’s data file during in our analyses to ensure that our findings are representative of the population of pregnant women in Nigeria. After adjusting for the survey’s complex sampling design, the weighted size of our study sample was 3,311.

### Dependent variables

#### Happiness

Happiness is the self-assessment of one’s overall happiness or unhappiness (Boehm and Kubzansky, 2012). Happiness was measured using the question “*taking all things together, would you say you are very happy, somewhat happy, neither happy nor unhappy, somewhat unhappy or very unhappy?*” As in a previous study by Inaba et al. (2015), happiness was operationalized as a dichotomous variable categorized as happy (responses with very happy or somewhat happy) and unhappy (responses with somewhat unhappy, neither happy nor unhappy or very unhappy).

#### Life satisfaction

LS was measured using the Cantril’s Self-Anchoring Ladder Life Satisfaction scale based on the question “*Now, look at this ladder with steps numbered from 0 at the bottom to 10 at the top. Suppose we say that the top of the ladder represents the best possible life for you and the bottom of the ladder represents the worst possible life for you. On which step of the ladder do you feel you stand at this time?*” Higher values reflected better LS. Similar to a previous study by Emerson and Llewellyn (2023), we modeled LS as binary outcome with low LS defined as a score of 5 or below while high LS was defined as a score of 6–10.

#### Optimism

Optimism describes the positive expectations of an individual about the future (Diener et al., 2017). We measured optimism using a single question “*In one year from now, do you expect that your life will be better, will be more or less the same, or will be worse, overall?*” Optimism was modeled as a dichotomous variable and categorized as high (will be better) and diminished (will be more or less the same, or will be worse, overall) similar to the approach followed by Lim et al. (2017).

### Independent variable

The primary exposure of interest in this analysis was pregnancy intention. We determined women’s pregnancy intention using the conventional timing-based measure, derived from responses to three questions contained in the MICS6. First, participants were asked, “*Are you pregnant now?”* Those responding affirmatively were asked, “*When you got pregnant, did you want to get pregnant at that time?*” with response options of “Yes” or “*No*”. Participants responding affirmatively were categorized as reporting a pregnancy as intended. Those who responded with “*No*” were further asked: “*Did you want to have a baby later on, or did you not want any more children*?” Those who indicated “*later*” were classified as reporting a mistimed pregnancy, while those who responded with “*none*” or “*no more*” were considered to be reporting an unwanted pregnancy. Both mistimed and unwanted pregnancies were considered as unintended.

### Confounding variables

The ecological systems theory (EST) provides the theoretical framework for which confounders were identified, selected and included in our analysis (Bronfenbrenner, 1977). According to this theory, individuals’ behaviors and well-being are influenced by interrelationships of multiple factors within and across different levels of their environment (McLeroy et al., 1988; Brothers et al., 2020; Janssen et al., 2022). These levels include intrapersonal-, interpersonal-, community- or organizational- and public policy levels. The covariates were identified a priori following a comprehensive literature review (Hardee et al., 2004; Maxson and Miranda, 2011; Yeatman and Smith-Greenaway, 2018), and were subsequently included in the analyses based on their conceptual importance, their biologic plausibility in the relationship between pregnancy intention and SWB and their availability in the survey data.

We categorized the covariates into individual-/household- and community-level characteristics. The individual-level characteristics include maternal age (15–24 years, 25–34 years, 35–49 years), married or cohabiting (yes, no), parity (none = 0, low = 1–2, medium = 3–4, high = 5), household wealth index (low, medium, high), religion (Christian, Non-Christian), health insurance coverage (covered, not covered). Community-level factors include place of residence (rural, urban) and geographic region of residence (North Central, North East, North West, South East, South South, South West).

### Statistical analyses

All analyses were conducted using SAS version 9.4 (SAS Institute Inc.) while data visualization was performed using R version 4.3.2 (R Project for Statistical Computing). Further, all analyses were weighted in order to generate robust estimates of the standard errors. For descriptive analyses, we used the PROC SURVEYMEANS command to estimate weighted means (SE) for continuous variables while the PROC SURVEYFREQ command was used to estimate weighted frequencies (%) for categorical variables. Further, Rao-Scott chi-square test was used to investigate differences between each measure of SWB and individual-/household- and community-level variables.

#### Multilevel model building strategy

Considering that the MICS has a hierarchical data structure, with women (*i*) nested within communities (*j*), and the dichotomous nature of the dependent variable, we fit a series of two-level logistic regression models with random intercepts. These models were adjusted for both individual-level (Level 1) and community-level (Level 2) factors. We used SAS PROC GLIMMIX with a binomial distribution and the LOGIT link function. All models were estimated using the pseudo-maximum likelihood approach with adaptive quadrature.

First, we defined a null model, excluding Level 1 and Level 2 predictors, to assess between-community variation across SWB measures. Subsequently, we developed more complex conditional models. Model I included the main exposure variable, Model II included Model I and adjusted for individual-level factors, while Model III incorporated Model I and adjusted for community-level variables. Lastly, Model IV included Model I with adjustments for both individual and community-level factors. Fixed effects were represented as odds ratios (ORs) along with their corresponding 95% confidence intervals (CIs). All tests were two-tailed and *p*-values <0.05 were considered to be statistically significant.

We assessed random effects for the measures of SWB using the intraclass correlation coefficient (ICC), median odds ratio (MOR) and proportional change in variance (PCV). The ICC quantifies the proportion of total observed variability in SWB measures that can be attributed to between-community variability (Austin and Merlo, 2017). The MOR measures the variability between communities by comparing two individuals randomly selected from different communities while the PCV estimates changes in cluster-level variability relative to the null model after adjusting for individual-/household-level and community-level characteristics. The goodness of fit of different models were assessed using the Akaike Information Criterion (AIC). Smaller AIC values indicated better fit.

## Results

### Sample characteristics

Table 1 reports weighted descriptive statistics for the study sample. The mean (SE) age of the sample was 28.05 (0.44) years, nearly one-half were between 25 and 34 years, most were either married or cohabiting (95.61%) and had no health insurance coverage (97.71%). Further, at least one-third of the sample were of low parity (33.71%) and identified as Christians (34.59%). Approximately one-half of the sample population resided in poor households (49.22%), two-thirds resided in rural areas (66.93%) and one-third of resided in the southern region of the country (30.04%).

**Table 1.** Weighted distribution of pregnancy intention status by individual and community-level characteristics, Nigeria, MICS 2021, *N* = 3,491

			Pregnancy intention						
	Total		Intended		Mistimed		Unwanted		
Variables	*N*	%	*N*	%	*N*	%	*N*	%	*p*-value
Overall	3,491	100	2,748	78.76	569	15.88	174	5.35	<0.0001
Individual-level characteristics									
Maternal age, years									
15–24	1,035	28.73	839	80.57	181	18.17	15	1.26	<0.0001
25–34	1,588	46.12	1,260	80.00	272	16.34	56	3.65	
35–49	868	25.15	649	74.41	116	12.44	103	13.15	
Marital status									
Single	110	3.39	45	43.56	56	47.24	9	9.19	<0.0001
Married/cohabiting	3,381	95.61	2,703	79.99	513	14.79	165	5.22	
Parity									
None (0)	522	15.03	421	84.57	96	14.74	5	0.68	<0.0001
Low (1–2)	1,150	33.71	977	83.46	162	15.90	11	0.64	
Medium (3–4)	900	24.98	699	75.99	153	16.55	48	7.46	
High (5+)	919	26.27	651	72.05	158	15.89	110	12.06	
Religious affiliation									
Christian	1,138	34.59	890	79.85	184	15.88	64	4.27	0.37
Non-Christian	2,353	65.41	1,858	78.19	385	15.89	110	5.92	
Health insurance coverage									
Not covered	3,435	97.71	2,712	78.87	551	15.67	172	5.46	0.09
Covered	56	2.29	36	74.08	18	25.28	2	0.64	
Household wealth index									
Low	1,927	49.22	1,521	79.39	314	16.01	92	4.59	0.74
Average	722	19.77	560	78.26	128	16.29	34	5.45	
High	842	31.01	667	78.09	127	14.43	48	6.48	
Community-level characteristics									
Place of residence									
Rural	2,597	66.93	2,054	79.46	422	15.85	121	4.68	0.34
Urban	894	33.07	694	77.34	147	15.95	53	6.71	
Geographic region									
North Central	657	14.57	514	78.8	100	15.46	43	5.72	0.38
North East	865	17.49	700	79.89	133	15.86	32	4.25	
North West	1,196	37.87	943	78.89	199	15.74	54	5.36	
South East	238	7.81	189	84.48	34	12.54	15	2.98	
South South	298	10.76	210	69.91	72	22.36	16	7.74	
South West	237	11.49	192	80/93	31	13.15	14	5.92	

*Note*: Frequencies, *N* are unweighted while percentages, % are weighted using the survey sampling weights to account for the complex sampling design of the MICS. All percentages reported are row percentages.

Abbreviation: SE, standard error.

The characteristics of the sample and stratified by pregnancy intention categories are shown in Table 1. Of the 3,419 pregnant Nigerian women surveyed, 743 (21.24%, 95% CI = 19.09%–23.38%) women reported their pregnancy was unintended, with 569 (15.88%, 95% CI = 14.12%–17.65%) and 174 (5.35%, 95% CI = 4.12%–6.59%) reported as mistimed and unwanted, respectively. As shown in Table 1 also, a significant bivariate association was observed between pregnancy intention and a number of categorical variables (*P*s < 0.05). About 13% of respondents aged 35–49 years reported that their pregnancy was unwanted compared with 1.3% of respondents aged 15–24 years. Furthermore, nearly one in 10 non-partnered respondents reported their pregnancy unwanted compared to 5% of respondents who were partnered. Approximately 12% of respondents who were of high parity compared with 0.68% who were nulliparous, 0.64% who were of low parity and 7.5% who were medium parity reported their pregnancy was unwanted.

**Figure 1.**
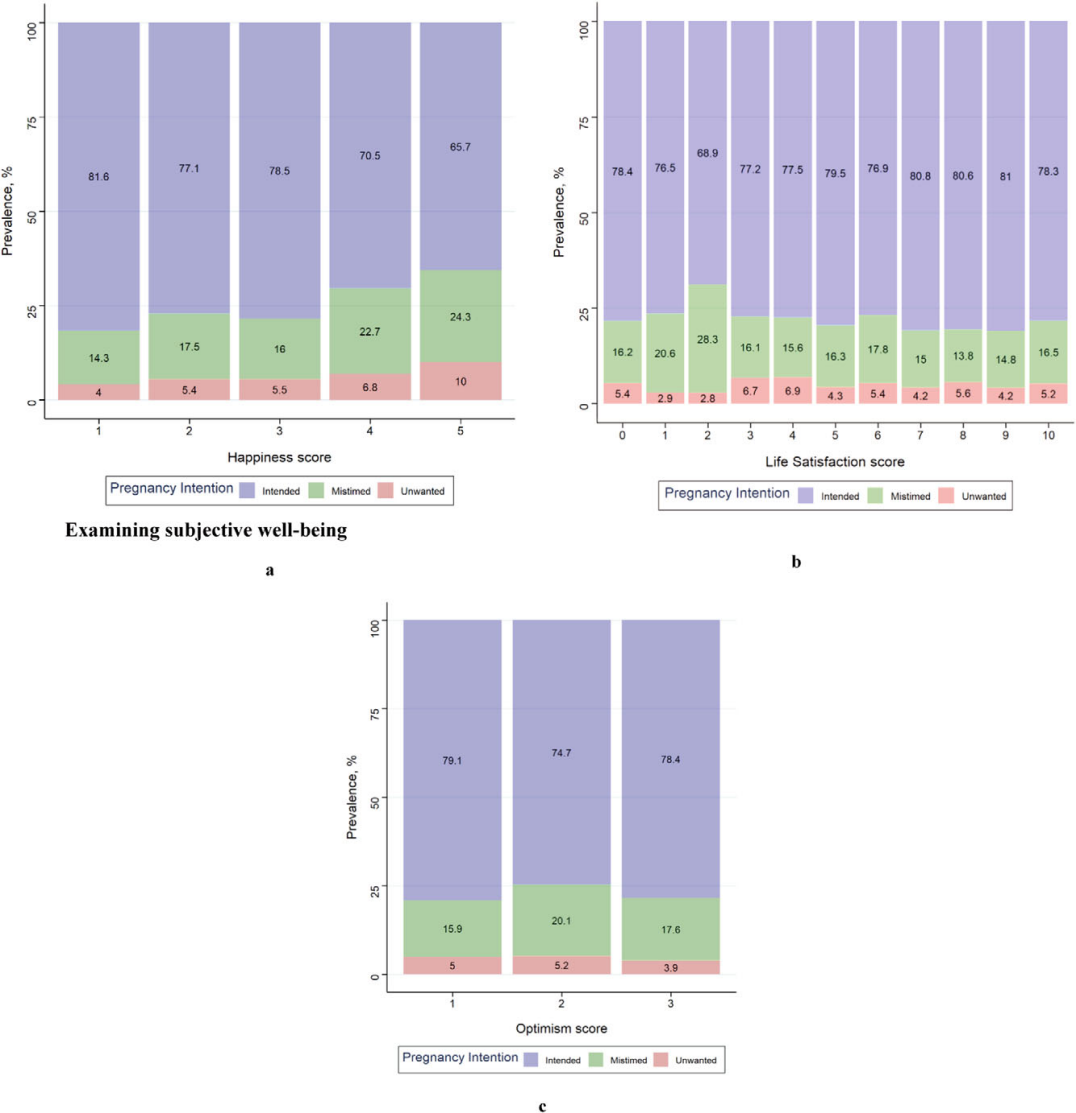
Distribution of pregnancy intention across self-reported (**a**) happiness, (**b**) life satisfaction and (**c**) optimism scores. Note that for happiness, 1 indicates very happy and 5 indicates very unhappy. For life satisfaction, 0 indicates worst and 10 indicates best possible life. For optimism, 1 indicates better and 3 indicates worse.

**Figure 2.**
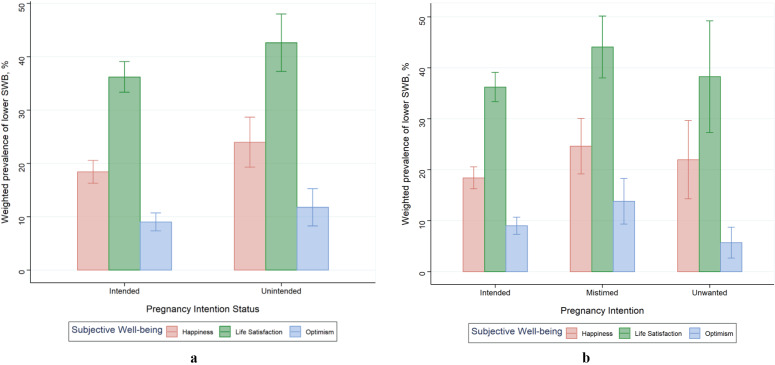
Prevalence of low SWB (weighted) according to pregnancy intention as a (**a**) two-category and (**b**) three-category variable among pregnant women 15–49 years. Error bars represent 95% confidence intervals.

The mean (SE) happiness, LS and optimism scores for the overall sample were 1.82 (0.03), 6.39 (0.06) and 1.11 (0.01), respectively. Table 2 shows the mean (SE) scores for each SWB measure stratified by the pregnancy intention categories. Figure 1 also shows the distribution of pregnancy intention across the different measures of SWB scores. Overall, at least 19.59% of the sample reported being unhappy, 37.58% reported experiencing low LS while 9.59% reported having diminished optimism. The prevalence of low SWB measures by pregnancy intention status are presented in Figure 2. There were significant differences in being unhappy (*p* = 0.016) and having diminished optimism (*p* = 0.008) levels but not for experiencing low LS (*p* = 0.069) across the three-category pregnancy intention variable. For the two-category pregnancy intention variable, there were significant differences in being unhappy (*p* = 0.045) and experiencing low LS (*p* = 0.033) but not for having diminished optimism (*p* = 0.137).

**Table 2.** Descriptive statistics (weighted) of scores for the different measures of SWB stratified by pregnancy intention status

	Pregnancy intention					
	Intended		Mistimed		Unwanted	
Subjective well-being measure	Mean	*SE*	Mean	*SE*	Mean	*SE*
Happiness scores	1.77	0.03	2.03	0.07	1.90	0.11
Life satisfaction scores	6.47	0.06	6.04	0.15	6.31	0.20
Optimism scores	1.11	0.01	1.17	0.03	1.07	0.02

### Multilevel analysis

#### Happiness

Table 3 presents estimates of the multilevel logistic regression models. In a typical community, where the random effect on the logit scale is zero, the estimated intercept was −1.91 indicating that the probability of being unhappy levels was 0.13. This probability varied significantly across communities levels across communities [*τ*_00_ = 2.284, *z*(1315) = 6.78, *p* < 0.0001]. The ICC was estimated to be 0.403 (40.3% of the total variability in the odds of being unhappy was attributed to differences across communities). The corresponding MOR was 4.15, suggesting a high variability in the odds of being unhappy levels. For example, the MOR indicates that pregnant women in the same community would have a median of more than four times the odds between different communities. The change in variance from Model IV (full model) demonstrates inclusion of community and individual-/household-level characteristics explained 2.19% of the variation in being unhappy. Based on the AIC values, the fully adjusted model (Table 3, Model IV) had the best fit for the data. Adjusting for individual-/household- and community-level factors explained a substantial amount of cluster-level variation. In terms of fixed effects, this model revealed that pregnant women with unintended pregnancy – either mistimed or unwanted – were no more or less likely to be unhappy compared to their counterparts whose pregnancies were intended (mistimed: aOR = 1.19, 95% CI = 0.88–1.60; unwanted: aOR = 1.16, 95% CI = 0.71–1.91) (Table 3, Model IV).

**Table 3.** Results from the multilevel logistic regression analyses investigating the association between maternal pregnancy intention and happiness, adjusting for individual-/household- and contextual-level factors among women in Nigeria, *N* = 3,491

	Null model^a^		Model I^b^		Model II^c^		Model III^d^		Model IV^e^	
Variables			OR	95% CI	aOR	95% CI	aOR	95% CI	aOR	95% CI
Fixed effects										
Intercept^†^	−1.911***		−1.982***		−0.76*		−1.918***		−0.636	
Individual-level characteristics										
Pregnancy intention status										
Intended			Ref		Ref		Ref		Ref	
Mistimed			**1.34**	1.01–1.79*	1.19	0.88–1.59	**1.34**	1.00–1.79*	1.19	0.88–1.60
Unwanted			1.48	0.93–2.37	1.18	0.72–1.93	1.48	0.93–2.36	1.16	0.71–1.91
Maternal age, years										
15–24					Ref				Ref	
25–34					1.24	0.92–1.68			1.23	0.91–1.66
35–49					1.41	0.97–2.05			1.44	0.99–2.09
Marital status										
Single					Ref				Ref	
Married/cohabiting					**0.25**	0.14–0.46***			**0.26**	0.14–0.48***
Parity										
None					Ref				Ref	
Low					1.09	0.75–1.59			1.09	0.75–1.59
Medium					1.01	0.66–1.54			1.01	0.66–1.54
High					1.38	0.87–2.19			1.34	0.84–2.13
Religious affiliation										
Non-Christian					Ref				Ref	
Christian					0.83	0.62–1.10			**0.68**	0.47–0.99*
Health insurance coverage										
Not covered					Ref				Ref	
Covered					0.48	0.14–1.60			0.49	0.15–1.59
Household wealth index										
Low					Ref				Ref	
Average					1.02	0.78–1.35			1.06	0.79–1.41
High					**0.67**	**0.48–0.93**			0.74	0.50–1.09
Community-level characteristics										
Place of residence										
Rural							Ref		Ref	
Urban							**0.72**	0.52–0.99*	0.79	0.55–1.17
Geographic region										
North Central							Ref		Ref	
North East							0.66	0.43–1.01	0.57	0.37–0.89
North West							1.41	0.97–2.07	1.16	0.76–1.76
South East							1.54	0.93–2.54	1.74	0.99–3.06
South South							1.08	0.66–1.77	1.16	0.67–1.99
South West							0.69	0.38–1.26	0.69	0.37–1.28
Random effects	Null model		Model I		Model II		Model III		Model IV	
Cluster-level variance (SE)	2.23 (0.33)		2.22 (0.33)		2.26 (0.34)		2.14 (0.32)		2.18 (0.33)	
ICC (%)	40.38		40.34		40.69		39.42		39.85	
MOR	4.15		4.15		4.19		4.03		4.09	
PCV (%)	Reference		19.29		−1.30		3.94		2.19	
Model fit statistics										
AIC	3,325.65		3,323.39		3,295.10		3,307.48		3,283.05	

*Notes*: Estimation method = Pseudo-maximum likelihood; containment degrees of freedom; reference level = happy. All estimates are weighted for the survey’s complex sampling design. Boldface indicates statistically significant results at the 0.05 level.

Abbreviations: OR, odds ratio; aOR, adjusted odds ratio; CI, confidence interval; ICC, intraclass correlation coefficient; MOR, median odds ratio; PCV, proportional change in variance; AIC, Akaike Information Criteria.

a Null model unconditional model, baseline model without any predictor variables.

b Model I – includes the main explanatory variable (pregnancy intention).

c Model II – Model I adjusted for only individual-/household-level characteristics.

d Model III – Model I adjusted for only community-level characteristics.

e Model IV – Model I adjusted for individual-/household- and community-level characteristics (full model).

† Estimates presented as log odds.

Values in bold significant at *p* < 0.05; ***p < 0.001, **p < 0.01, *p < 0.05.

#### Life satisfaction

The results of the multilevel logistic regression for LS are presented in Table 4. The estimated intercept for the empty model (−0.578) (Table 4, null model) suggests that in a typical community, the probability of reporting low LS was 0.36 and varied significantly across communities [*τ*_00_ = 1.346, *z*(1315) = 6.92, *p* < 0.0001]. Approximately 29.03% of the total variability in the odds of reporting low LS was attributed to between-community differences (ICC = 0.290). The corresponding MOR was 3.02, suggesting a high variability in the odds of reporting low LS Incorporating both individual- and community-level characteristics showed a 2.98% decrease in the explained variance in the odds of reporting low LS relative to the null model. Based on the AIC values, the fully adjusted model (Table 4, Model IV) had the best fit for the data. In terms of fixed effects, the results show that after adjusting for individual-/household- and community-level characteristics, there was no statistically significant association between pregnancy intendedness and low LS (mistimed, aOR = 1.07, 0.83–1.37, unwanted, aOR = 1.06, 95% CI = 0.69–1.65) (Table 4, Model IV).

**Table 4.** Results from the multilevel logistic regression analyses investigating the association between maternal pregnancy intention and life satisfaction, adjusting for individual-/household- and contextual-level factors among women in Nigeria, *N* = 3,491

	Null model^a^	Model I^b^		Model II^c^		Model III^d^		Model IV^e^	
Variables		OR	95% CI	aOR	95% CI	aOR	95% CI	aOR	95% CI
Fixed effects									
Intercept^†^	−0.578***	−0.009***		0.492		−0.695***		0.333	
Individual-level characteristics									
Pregnancy intention status									
Intended		Ref		Ref					
Mistimed		1.16	0.91–1.47	1.07	0.83–1.36	1.16	0.91–1.48	1.07	0.83–1.37
Unwanted		1.15	0.76–1.74	1.04	0.67–1.61	1.18	0.78–1.79	1.06	0.69–1.65
Maternal age, years									
15–24				Ref				Ref	
25–34				1.08	0.85–1.36			1.11	0.87–1.39
35–49				1.09	0.79–1.45			1.11	0.82–1.50
Marital status									
Single				Ref				Ref	
Married/cohabiting				**0.35**	0.21–0.59**			**0.34**	0.20–0.57***
Parity									
None				Ref				Ref	
Low				1.06	0.80–1.41			1.07	0.80–1.41
Medium				1.00	0.72–1.39			0.98	0.71–1.36
High				1.22	0.85–1.74			1.17	0.82–1.69
Religious affiliation									
Non-Christian				Ref				Ref	
Christian				0.87	0.69–1.08			1.01	0.75–1.36
Health insurance coverage									
Not covered				Ref				Ref	
Covered				**0.45**	0.20–0.98*			**0.44**	0.19–0.96*
Household wealth index									
Low				Ref				Ref	
Average				0.86	0.68–1.08			0.87	0.68–1.11
High				**0.65**	0.51–0.84**			**0.66**	0.49–0.89**
Community-level characteristics									
Place of residence									
Rural						Ref		Ref	
Urban						0.88	0.69–1.13	1.12	0.83–1.51
Geographic region									
North Central						Ref		Ref	
North East						1.36	0.98–1.89	1.33	0.94–1.88
North West						1.26	0.94–1.69	1.23	0.88–1.72
South East						1.03	0.66–1.59	1.03	0.64–1.67
South South						0.96	0.65–1.41	0.93	0.61–1.43
South West						0.81	0.52–1.27	0.78	0.49–1.23
Random effects	Null model	Model I		Model II		Model III		Model IV	
Cluster-level variance (SE)	1.34 (0.19)	1.35 (0.19)		1.41 (0.20)		1.33 (0.19)		1.39 (0.20)	
ICC (%)	29.03	29.07		29.96		28.85		29.64	
MOR	3.02	3.02		3.10		3.01		3.07	
PCV (%)	Reference	−0.22		−4.58		0.89		−2.98	
Model fit statistics									
AIC	4,514.45	4,516.52		4,494.46		4,516.82		4,500.19	

*Notes*: Estimation method = pseudo-maximum likelihood; containment degrees of freedom; reference level = low life satisfaction. All estimates are weighted for the survey’s complex sampling design. Boldface indicates statistically significant results at the 0.05 level.

Abbreviations: OR, odds ratio; aOR, adjusted odds ratio; CI, confidence interval; ICC, intraclass correlation coefficient; MOR, median odds ratio; PCV, proportional change in variance; AIC, Akaike Information Criteria.

a Null model unconditional model, baseline model without any predictor variables.

b Model I – includes the main explanatory variable (pregnancy intention).

c Model II – Model I adjusted for only individual-/household-level characteristics.

d Model III – Model I adjusted for only community-level characteristics.

e Model IV – Model I adjusted for individual-/household- and community-level characteristics (full model).

† Estimates presented as log odds.

Values in bold significant at *p* < 0.05; ***p < 0.001, **p < 0.01, *p < 0.05.

#### Optimism

Table 5 presents estimates of the multilevel logistic regression model for optimism. The estimated intercept for the empty model was −3.10. This suggests that in a typical community, the probability of reporting worse optimism was 0.05. This probability varied across communities [*τ*_00_ = 2.580, *z*(1315) = 6.01, *p* < 0.0001]. The ICC was estimated to be 0.439, suggesting that 43.9% of the total variability in the odds of reporting diminished optimism was due to systematic differences between communities. Correspondingly, the MOR was 4.63, suggesting a high variability in the odds of reporting worse optimism levels. The change in variance in the full model in Table 5 showed that approximately 33% of the variation in reporting worse optimism was explained by incorporating individual-/household- and community-level characteristics. While the difference in AIC values between Model III and Model IV is minimal, the 5.71 increase is in Raftery’s range (O’Connell and McCoach, 2008), thus, favoring the more complex model (Model IV). Based on this model, there also appears to be no relationship between women’s pregnancy intention and reporting worse optimism, after adjusting for individual and community-level factors (mistimed: aOR = 1.22, 95% CI = 0.82–1.82; unwanted: aOR = 1.07, 95% CI = 0.56–2.04) (Table 5, Model IV).

**Table 5.** Results from the multilevel logistic regression analyses investigating the association between maternal pregnancy intention and optimism, adjusting for individual-/household- and contextual-level factors among women in Nigeria, *N* = 3,479

	Null Model^a^	Model I^b^		Model II^c^		Model III^d^		Model IV^e^	
Variables		OR	95% CI	aOR	95% CI	aOR	95% CI	aOR	95% CI
Fixed effects									
Intercept^†^	−3.108***	−3.159***		−2.267***		−3.781***		−2.807***	
Individual-level characteristics									
Pregnancy intention status									
Intended		Ref		Ref				Ref	
Mistimed		1.31	0.87–1.87	1.18	0.79–1.77			1.22	0.82–1.82
Unwanted		1.07	0.57–2.00	1.01	0.53–1.94			1.07	0.56–2.04
Maternal age, y									
15–24				Ref				Ref	
25–34				0.83	0.57–1.21			0.87	0.59–1.27
35–49				0.65	0.39–1.07			0.67	0.40–1.12
Marital status									
Single				Ref				Ref	
Married/ cohabiting				1.19	0.74–1.90			**0.37**	0.17–0.81**
Parity									
None				Ref				Ref	
Low				1.18	0.74–1.90			1.24	0.77–1.98
Medium				**1.77**	1.05–2.99*			1.75	1.04–2.95
High				1.67	0.93–2.99			1.60	0.89–2.85
Religious affiliation									
Non-Christian				Ref				Ref	
Christian				**0.64**	0.44–0.92*			0.75	0.46–1.22
Health insurance coverage									
Not covered				Ref				Ref	
Covered				0.83	0.31–2.25			0.85	0.33–2.21
Household wealth index									
Low				Ref				Ref	
Average				0.96	0.65–1.41			0.98	0.65–1.48
High				0.80	0.53–1.22			0.81	0.49–1.34
***Community-*** ***level***									
Residence									
Rural						Ref		Ref	
Urban						1.09	0.75–1.62	1.23	0.78–1.94
Region									
North Central						Ref		Ref	
North East						**4.29**	2.53–7.32***	**3.84**	2.21–6.68***
North West						**2.04**	1.22–3.45**	1.74	0.98–3.06
South East						**4.36**	2.36–8.04***	**5.12**	2.60–10.09***
South South						**0.37**	0.15–0.94*	0.41	0.15–1.11
South West						0.81	0.35–1.90	0.83	0.35–1.99
Random effects	Null model	Model I		Model II		Model III		Model IV	
Cluster-level variance (SE)	2.58 (0.43)	2.58 (0.43)		2.57 (0.46)		2.15 (0.38)		2.14 (0.38)	
ICC (%)	43.95	43.93		43.85		39.51		39.58	
MOR	4.63	4.63		4.61		4.05		4.04	
PCV	Reference	−0.22		0.76		31.99		32.58	
Model fit statistics									
AIC	2,205.24	2,206.88		2,205.91		2,148.38		2,154.09	

*Notes*: Estimation method = pseudo-maximum likelihood; containment degrees of freedom; reference level = better optimism. All estimates are weighted for the survey’s complex sampling design. Boldface indicates statistically significant results at the 0.05 level.

Abbreviations: OR, odds ratio; aOR, adjusted odds ratio; CI, confidence interval; ICC, intraclass correlation coefficient; MOR, median odds ratio; PCV, proportional change in variance; AIC, Akaike Information Criteria.

a Null model unconditional model, baseline model without any predictor variables.

b Model I – includes the main explanatory variable (pregnancy intention).

c Model II – Model I adjusted for only individual-/household-level characteristics.

d Model III – Model I adjusted for only community-level characteristics.

e Model IV – Model I adjusted for individual-/household- and community-level characteristics (full model).

† Estimates presented as log odds.

Values in bold significant at *p* < 0.05; ***p < 0.001, **p < 0.01, *p < 0.05.

## Discussion

Current evidence on the association between pregnancy intendedness and low SWB measures remains limited (Hill et al., 2019). In this study, we aimed to address this gap by investigating the relationship between pregnancy intendedness and low SWB measures using cross-sectional population-level data. To account for the methodological challenge of data clustering within higher-level units, we employed a multilevel modeling approach. This allowed us to explore how SWB outcomes are influenced by higher-level contextual factors. Our findings reveal that approximately one in five pregnant women in the sample reported feeling unhappy, nearly 40% reported experiencing low LS and one in 10 reported having diminished optimism. While the odds of reporting low levels of SWB measures varied substantially across communities, our overall analysis revealed that pregnant women reporting either mistimed or unwanted pregnancy were just as likely to report unhappiness, low LS and diminished optimism as were those who reported intended pregnancy, contrary to our initial hypothesis. These results appear to be consistent with and contribute to the emerging body of literature emphasizing the need for understanding the link between pregnancy intention and subjective and psychological well-being. One study conducted among pregnant women in Iran did not show a significant association between pregnancy intention and happiness (Pishgar et al., 2016). Similarly, Yeatman and Smith-Greenaway (2018) demonstrated that pregnancy intention was not association with SWB among women in Malawi.

Our findings are at odds with those of several studies demonstrating that pregnancy intentions were associated with low SWB. For example, Ali et al. (2023) reported a two-fold higher odds of reporting higher happiness levels among women in the United Arab Emirates reporting planned pregnancy compared to those whose pregnancy was unplanned. Also, evidence from another study conducted in the United States suggests that women who reported having unintended births experienced decreased happiness levels relative to women with no children (Su, 2012). More broadly, Hardee et al. (2004) have demonstrated a significant a negative relationship between unintended pregnancy and psychological well-being among women in Indonesia. Taken together, the above findings from previous research, in addition to the results of our study, indicate that existing evidence regarding the association between pregnancy intendedness and SWB among pregnant women remains inconclusive at best, thereby warranting the need for further empirical research.

In contrast to our study, prior studies not only failed to establish a distinction between the subcategories of unintended pregnancy but also employed different methods for measuring and operationalizing SWB. These variations in methodological approaches could explain the substantial disparities across these results, even as our study leveraged data which captured different dimensions of SWB based on best practices in the field of SWB (économiques, 2013). Yeatman and Smith-Greenaway (2018) argue, based on the results of their study, that the absence of compelling evidence linking pregnancy intention to SWB could be attributed to two factors. First, SWB may remain relatively unaffected by a woman’s pregnancy intention. Second, the prospect of motherhood itself could act as a buffer, mitigating any potential adverse effects that pregnancy intention might have on SWB. In our case, we contend that our estimates might have been subject to residual confounding, such that factors not accounted for, including education and gestational age, either due to their unavailability or substantial missing data – could have influenced the outcomes and conclusions drawn from our research. Furthermore, we believe that since the cognitive and affective evaluations of one’s life are not stable, it may well be challenging to detect significant differences based solely on cross-sectional data, as was the case in our study. For example, research has shown that LS increased from pregnancy to postpartum (Dyrdal et al., 2011; Gebuza et al., 2014). More recent evidence also revealed racial disparity in LS, with LS increasing over the perinatal period for White but not Black American women. Though these studies did not account for pregnancy intention, the findings underscore the importance of recognizing the temporal changes in SWB and, by extension, the valuable role that prospective longitudinal research can play in providing a comprehensive perspective on how pregnancy intention relates to SWB.

Moreover, the inconsistent results highlight an important gap in studies of SWB where varied approaches have been used to capture measures of SWB. Nonetheless, our study is more likely to provide reliable and consistent estimates that can be compared across different contexts, and with emerging studies in this area, especially in relation to the sustainable development agenda. This is predicated on our use of robust research methods and analytical techniques, such as multilevel regression modeling and adjustment for potential confounding variables. Furthermore, we used established conventions for operationalizing pregnancy intendedness and SWB, increasing the comparability of its results across different populations and settings. Additionally, the design and methodology employed in our study make it suitable for cross-national comparisons, enabling researchers to evaluate similarities and differences in the association between pregnancy intendedness and SWB across diverse cultural, social and economic contexts. Lastly, the objectives and findings of our study are relevant to broader global initiatives aimed at promoting well-being and achieving sustainable development goals, as captured in SDG 3. This alignment ultimately improves the significance of our study and the potential implications for maternal and reproductive health policy and practice.

Although the results from the fully adjusted multivariable models revealed significant associations between several individual/household and community-level characteristics and low SWB measures, we refrained from interpreting these effect estimates to avoid the “Table 2 fallacy”, which occurs when effect estimates of confounders in an epidemiological model are erroneously interpreted as total-effect estimates of their association with the outcome, when indeed the model was built to estimate the total effect of the primary independent variable (Westreich and Greenland, 2013; Auerbacher et al., 2023; van Zwieten et al., 2024).

## Implications for research and practice

In addition to the important role of longitudinal studies in this field, qualitative research methods also present a unique opportunity for exploring other relational and contextual factors that could influence the relationship between pregnancy intention and SWB. Hence, combining both approaches can improve our understanding and lead to more effective interventions and support for women experiencing declines in SWB during pregnancy. Furthermore, to enhance comparability of findings across different contexts, there is need to develop standardized and robust measures of SWB. Prenatal health providers should also consider incorporating routine screening for pregnancy intendedness and assess women’s SWB during preconception, prenatal and postpartum visits. This practice has the potential to identify women at risk of low SWB and could substantially improve the quality of care and support offered to both expectant and new mothers.

## Strengths and limitations

In our analysis, we differentiated between the categories of mistimed and unwanted pregnancy. By doing so, we disentangle the differences in effects on the various dimensions of SWB. Unlike in previous studies where a single measure was used as a proxy to investigate SWB, we employed measures across the different domains of SWB in order to provide a robust assessment of SWB, avoid oversimplification, reduce bias and gain a more nuanced understanding of its association with unintended pregnancy. By using nationally representative data, our study provides estimates that are robust and generalizable which ultimately are relevant for informing policies and interventions aimed at improving the well-being among the population of pregnant women.

There were several limitations of our study. First, maternal pregnancy intention was captured using self-reports of retrospective recollection of preconception pregnancy desire. A generally cited limitation of this using this approach is that reports may be influenced by *ex post* rationalization as well as recall bias (Blondel et al., 2023; Zimmerman et al., 2023). Second, the findings of our study should be interpreted with caution in view of the cross-sectional nature of the data used. As earlier stated, the temporal nature of SWB precludes the extent to which the conclusions drawn from our study are generalizable. Nonetheless, our study lays a foundation upon which future research can build on. Thirdly, while concerns may arise regarding the utility of single items in capturing the various measures of SWB due to the complexity of the constructs, existing literature provides evidence supporting this approach. Single items have demonstrated comparable performance to multiple-item scales in measuring SWB (Prati, 2022). Finally, the MICS survey lacked relevant obstetric variables, including gestational age, which precluded our ability to adjust for these important confounders in our models. Gestational age is likely to influence women’s pregnancy desirability and SWB. Therefore, the lack of this variable is likely to result in biased estimates.

## Conclusions

Our study provides substantial conceptual and methodological contributions to the current literature by examining the relationship between pregnancy intention and low SWB. Overall, cross-sectional evidence from our study suggesting unintended pregnancy adversely impacts happiness, LS and optimism levels among pregnant women in Nigeria is inconclusive. Further research using standardized measures of variable ascertainment, as in our study, is warranted to strengthen the evidence base of the association between SWB and pregnancy-related factors.

## Data Availability

The survey data used for this study are publicly available from the 2021 Multiple Indicator Cluster Survey (MICS 6) and can be accessed from https://mics.unicef.org/surveys.
