# Supplementary material for: Examining subjective well-being during pregnancy and its association with pregnancy intendedness among women in Nigeria: A population-based cross-sectional multilevel study
Source: Glob Ment Health (Camb). 2024 Oct 21;11:e87. doi: 10.1017/gmh.2024.99 (PMC11504940; doi:10.1017/gmh.2024.99)
Supplement: Peer review history

# Editor report: Recommendation: Examining subjective well-being during pregnancy and its association with pregnancy intendedness among women in Nigeria: A population-based cross-sectional multilevel study — R0/PR2

**DOI:** 10.1017/gmh.2024.99.pr2

**Affiliations:** Fudan University, China

No accompanying comment.

# Editor report: Decision: Examining subjective well-being during pregnancy and its association with pregnancy intendedness among women in Nigeria: A population-based cross-sectional multilevel study — R0/PR3

**DOI:** 10.1017/gmh.2024.99.pr3

**Affiliations:** London School of Hygiene & Tropical Medicine, United Kingdom of Great Britain and Northern Ireland

No accompanying comment.

# Editor report: Recommendation: Examining subjective well-being during pregnancy and its association with pregnancy intendedness among women in Nigeria: A population-based cross-sectional multilevel study — R1/PR5

**DOI:** 10.1017/gmh.2024.99.pr5

**Affiliations:** Fudan University, China

No accompanying comment.

# Editor report: Decision: Examining subjective well-being during pregnancy and its association with pregnancy intendedness among women in Nigeria: A population-based cross-sectional multilevel study — R1/PR6

**DOI:** 10.1017/gmh.2024.99.pr6

**Affiliations:** London School of Hygiene & Tropical Medicine, United Kingdom of Great Britain and Northern Ireland

No accompanying comment.

# Editor report: Recommendation: Examining subjective well-being during pregnancy and its association with pregnancy intendedness among women in Nigeria: A population-based cross-sectional multilevel study — R2/PR8

**DOI:** 10.1017/gmh.2024.99.pr8

**Affiliations:** Fudan University, China

No accompanying comment.

# Editor report: Decision: Examining subjective well-being during pregnancy and its association with pregnancy intendedness among women in Nigeria: A population-based cross-sectional multilevel study — R2/PR9

**DOI:** 10.1017/gmh.2024.99.pr9

**Affiliations:** London School of Hygiene & Tropical Medicine, United Kingdom of Great Britain and Northern Ireland

No accompanying comment.
